# Comparison of the Neutrophil Proteome in Trauma Patients and Normal Controls

**DOI:** 10.2174/092986612800493977

**Published:** 2012-06

**Authors:** Liz M.B. Teles, Elaine N. Aquino, Anne C.D. Neves, Carlos H.S. Garcia, Peter Roepstorff, Belchor Fontes, Mariana S. Castro, Wagner Fontes

**Affiliations:** a Laboratory of Biochemistry and Protein Chemistry, Cell Biology Department, University of Brasília, Brazil; b Department of Biochemistry and Molecular Biology, University of Southern Denmark, Odense, Denmark; c Laboratory of Medical Investigation (LIM-62), 3rd Division of Clinical Surgery, Hospital das Clínicas, University of São Paulo School of Medicine, Brazil; d Laboratory of Toxinology, Physiological Sciences Department, University of Brasília, Brazil

**Keywords:** Neutrophils, trauma, proteome, systemic inflammatory response syndrome, inflammation.

## Abstract

Background: Neutrophils have an impressive array of microbicidal weapons, and in the presence of a pathogen, progress from a quiescent state in the bloodstream to a completely activated state. Failure to regulate this activation, for example, when the blood is flooded with cytokines after severe trauma, causes inappropriate neutrophil activation that paradoxically, is associated with tissue and organ damage. Acidic proteomic maps of quiescent human neutrophils were analyzed and compared to those of activated neutrophils from severe trauma patients. The analysis revealed 114 spots whose measured volumes differed between activated and quiescent neutrophils, with 27 upregulated and 87 downregulated in trauma conditions. Among the identified proteins, grancalcin, S100-A9 and CACNB2 reinforce observed correlations between motility and ion flux, ANXA3, SNAP, FGD1 and Zfyve19 are involved in vesicular transport and exocytosis, and GSTP1, HSPA1 HSPA1L, MAOB, UCH-L5, and PPA1 presented evidence that activated neutrophils may have diminished protection against oxidative damage and are prone to apoptosis. These are discussed, along with proteins involved in cytoskeleton reorganization, reactive oxygen species production, and ion flux. Proteins such as Zfyve19, MAOB and albumin- like protein were described for the first time in the neutrophil. In this work we achieved the identification of several proteins potentially involved in inflammatory signaling after trauma, as well as proteins described for the first time in neutrophils.
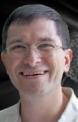

## INTRODUCTION

Trauma is major cause of mortality in the Western world, for people under the age of 45 [1-2]. The mechanical energy of severe trauma may injure tissues and organs, and usually results in intense bleeding and hemorrhagic shock, leading to tissue ischemia. Other insults, such as surgical interventions or infections, may aggravate the patient's condition [3-4]. Fluid resuscitation after prolonged shock involves reperfusion that carries inflammatory mediators from ischemic tissue to multiple organs, in which may lead to the development of the complex phenomenon of systemic inflammatory response syndrome (SIRS) and acute respiratory distress syndrome (ARDS). This trauma-induced immune response involves the activation of diverse cell types, mainly polymorphonuclear neutrophils which, besides controlling infection by phagocytosing bacteria, are well known for their apparently paradoxical role as an important cell mediator in the SIRS mechanism.

Compensatory anti-inflammatory response syndrome (CARS) is another response to trauma whose mechanism is not yet completely established. CARS results from the counter-regulating suppression of various cellular and humoral immune functions, and is characterized by an excessive production of anti-inflammatory mediators, such as interleukin-10 (IL-10) [5], and is associated with the onset of sepsis.

Neutrophils are the most extensively studied cells of the inflammatory response and are implicated in the pathogenesis of tissue injury after trauma. The function of neutrophils in host defense and injury has been the topic of intense investigation [6]. However, in spite of substantial advances in our understanding of the biochemistry of innate immune response cells such as neutrophils, few innovations have targeted the diagnostic indicators of the systemic acute inflammatory response to trauma or sepsis [7].

Neutrophils are activated by a variety of receptor-mediated agonists, and undergo rapid changes in phenotype and function, including adhesion, migration, phagocytosis, generation of reactive oxygen species (ROS), and degranulation of stored inflammatory proteins. These processes are performed and regulated by proteins in a complex interactive network. Activation mechanisms identified to date largely involve post-transcriptional checkpoints [8]. In addition to post-translational modifications, splicing and allelic variation can contribute to the tight control of the synthesis of cytokines, ion channels and metabolism-related proteins.

Currently, laboratory evaluation of patients with suspected sepsis or inflammatory disorders such as SIRS and ARDS uses tests with poor sensitivity and specificity. The complexity of the clinical presentation of SIRS, ARDS and sepsis requires new assays with early diagnostic and prognostic value. Possible candidates for these assays maybe found in neutrophil proteins, which are investigated in this work by comparative analysis of the proteomes of quiescent and trauma-activated cells.

## MATERIALS AND METHODS

### Sample Preparation

Neutrophils for the control group were obtained from volunteer donors with no history of chronic diseases, no recent inflammatory diseases, and no medication use within the last 60 days. Polymorphonuclear leukocytes were obtained from major torso trauma patients at the HC-FMUSP, 24-48 h after the traumatic event. Trauma patients had an anatomical injury severity score (ISS) of 29, received no more than 4 units of blood products transfusion up to the moment of sample collection [9], and presented no hollow viscera injury and no signs of infection. Donors and patients were 25-45 years old. The project was approved by the ethical committee of HC-FMUSP (process # 2001045) before donors and patients were contacted.

Neutrophils were isolated as described previously [10] with minor modifications. Briefly, blood was collected in heparinized solution and centrifuged through 60% and 70% density layers of Percoll (Amersham Biosciences, Uppsala, Sweden). Residual erythrocytes were removed by osmotic lysis. An aliquot was taken for cell counting in Neubauer chamber and viability analysis by nigrosine test. Purification was at room temperature to avoid activation, except for the density gradient step.

Samples yielding 2 x 10^7^ cells/mL (98% neutrophils, 99% viable) were immediately subjected to cell lysis in protein solubilization buffer (7 M urea, 2 M thiourea, 1% v/v Triton X-100, 0.5% v/v Pharmalyte 4-7, 65 mmol/L dithiotreitol - DTT) containing protease inhibitors. Cell lysis was in a shaker for 2 h at 25 °C. After protein solubilization, the supernatant following centrifugation of the cell lysate was frozen at -80 °C until electrophoretic separation.

### Two-Dimensional (2D) Polyacrylamide Gel Electrophoresis

Neutrophil protein extracts were submitted to protein quantification [11] and diluted in protein solubilization buffer, pH 4-7, to a final protein concentration of 0.14 μg/μL. A sample of 350 µL in lysis buffer was applied to 18-cm immobilized pH gradient (IPG) gel strips with a linear pH range of 4 to 7 (Amersham Biosciences, Uppsala, Sweden) and submitted to isoelectric focusing using an IPGphor (Amersham Biosciences, Uppsala, Sweden) [12]. After focusing, IPG gel strips were submitted to protein reduction in 125 mM DTT and alkylation in 135 mM iodoacetamide. Sodium dodecyl sulfate polyacrylamide gel electrophoresis (SDS-PAGE) was performed on 12% T gels run on a Protean II system (Bio-Rad, Hercules, CA, USA) at 25 mA per gel until the dye front reached the end. Proteins were visualized by ammoniacal silver staining, chosen for the improved sensitivity and compatibility to mass spectrometry [6, 13].

### Images and Statistical Analysis

Images from the total of 18 gels (nine gels of each group, comprising biological and technical replicates) were analyzed with ImageMaster Platinum software (Amersham Biosciences, Uppsala, Sweden). The experimental design was validated according to Chich *et al*. [14] Volumes for each spot were normalized against the total volume of the detected spots. For both the normal donors and the severe trauma patients, one gel was assigned as a standard for the remaining eight gels. After pairing within each group, the two master gels were matched. Paired spots were tested for normality of volumes using the Kolmogorov-Smirnov test. Significance of differences in spot volumes was assessed using an Independent-Samples T-test (for parametric samples) or Mann-Whitney test (for non-parametric samples). As noted by Biron and Magalhães *et al.* [15-16], two additional stringency criteria were applied. To avoid false positive results in statistical parameters, the difference in spot volume ratio was required to be at least twice between the groups of gels, and the spot was required to be present in at least five gels in each group. Spots were considered specifically present if they appeared in at least five gels in a group, and were not detected in any gels of the other group.

### Peptide Mass Fingerprinting (PMF) and Tandem Mass Spectrometry Fragmentation (MS/MS)

Silver-stained spots were excised, destained, in-gel digested with trypsin (Promega, Madison, USA), and extracted as previously described [17]. Before application on matrix-assisted laser desorption/ionization (MALDI) targets, digests were desalted using reversed-phase chromatography microcolumns and eluted on the surface of AnchorChip 384/600 targets (Bruker Daltonics, Leipzig, Germany) with a matrix mix of α-cyano-4-hydroxycinnamic acid and 2,5 dihydroxybenzoic acid, 1:1 (v/v), 5 µg/µL final concentration [18].

PMF used a MALDI-TOF/TOF mass spectrometer (Autoflex II; Bruker Daltonics, Leipzig, Germany), in positive and reflector mode, after external calibration using a standard peptide mixture ranging from 757 to 2465 mass units. Spectra were internally calibrated with peaks from keratin contaminant digestion and trypsin autolysis. Selected peaks were used in PMF searches for protein identification, and those that did not fulfill the validation criteria described below were subjected to MS/MS fragmentation. MS/MS fragmentation was carried out using the LIFT technique, which, combined with peptide mass fingerprinting, increased the identification rate [19]. Mass spectra were analyzed using FlexAnalysis and Biotools software (Bruker Daltonics). Protein identification used Mascot software. Searches were performed against the NCBInr database [20] with 50-100 ppm mass tolerance and a fragmentation mass tolerance of 90 mmu. Search parameters were restricted to *Homo sapiens* and one missed cleavage was allowed. Carbamidomethylation of cysteines was set as a fixed modification and oxidation of methionine and N-terminal acetylation as variable modifications.

To further validate Mascot results, we applied stringency criteria that the peptide mass error distribution should fit a distribution curve, and coherence between theoretical and experimental mass and pI values should be within 15%. Peptides containing missed cleavages and methionine oxidation were manually verified. To reduce redundancy and validate the sequence data, significant results (p <0.05) were analyzed in GPMAW software (Lighthouse data, Odense, Denmark) for multiple peptide location.

## RESULTS

An average of 850 spots were detected on each gel. After comparison and statistical analysis using the stringency criteria described above, 114 spots showed differential volumes, with 357 spots undetectable in activated neutrophils, and 244 spots exclusively found in activated neutrophils from severe trauma patients. Fig. (**1**) shows the 2D map for the quiescent neutrophil state highlighting the identified and the differentially expressed proteins. Table **1** summarizes information about each identified protein. In the section below, after each protein, the number in parenthesis indicates where the protein can be found in Table **1** and on Fig. (**1**).

Image of a 2D gel obtained from quiescent neutrophils. Spots whose measured volumes differed between the control and trauma conditions are in white. Boxes show examples of spot volume comparisons between the conditions. In the 3D view of the spot, the peak height represents staining intensity and the corresponding gel image is below. Numeric identification corresponds to the proteins in Table **1**.

The unique identified protein upregulated in activated neutrophils was PPA1 (1398) or pyrophosphatase enzyme. Proteins downregulated in trauma included ubiquitin carboxyl-terminal hydrolase L5 (UCH-L5) (1179), L-plastin (354), vimentin (521), β-actin (729), grancalcin (1282), S100-A9 (1543), α-soluble N-ethylmaleimide-sensitive factor (NSF)-attachment protein (SNAP) (1024), annexin A3 (ANXA3) (1052), heat shock 70 kDa protein 1 (HSPA1A) (358), glutathione S-transferase P (GSTP1) (1336), laminin receptor 1 (RSSA) (811), and Rho GDP dissociation inhibitor (RhoGDI) (1262) complex.

In the set of proteins detected only in quiescent cells, we identified KIAA0564 (71), histidyl-tRNA synthetase (HisRS) (526), interferon-induced protein with tetratricopeptide repeats 2 (IFIT2) (595), modulator of retrovirus infection (MRI) (1501), postmeiotic segregation increased 2-like 3 (PMS2L3) (1360), pseudouridylate synthase 7 homolog (*Saccharomyces cerevisiae*)-like (PUS7L) (1479), NCK-associated protein 1-like (NCKAP1L) (102), heat shock 70 kDa protein 1-like (HSPA1L) (508), desmin (603), albumin-like (679), zinc finger FYVE domain 19 (Zfyve19) (680), faciogenital dysplasia 1 protein (FGD1) - a protein reported as containing the domains FYVE, RhoGEF and PH (546), antibody-dependent cellular cytotoxicity (ADCC)-enhanced Fcγ fragment (629), monoamine oxidase type B (MAOB) (695), calcium channel voltage-dependent beta-2 subunit (CACNB2) (904) and chloride intracellular channel (CLIC4) (982).

## DISCUSSION

Adams and Botha *et al.* demonstrated that trauma modulates neutrophil function [21-22]. Our results showing differences in proteins in neutrophils from trauma patients and normal controls corroborate these findings. Of the 114 spots showing different volumes, 27 were upregulated and 87 were downregulated after activation by systemic inflammation caused by trauma. Previously, we identified 22 proteins [6] by 2D-acidic mapping of the quiescent neutrophil. In this article, 29 additional proteins were identified, enhancing the identification sensitivity of our previous study. The number of successful identifications was rather low because of contamination with keratin and low abundance in the gels. Among proteins differentially expressed between quiescent and activated neutrophils, we identified 1 upregulated and 12 downregulated proteins, and 16 that were below the detection level in polymorphonuclear leukocytes from trauma patients.

The identification of pyrophosphatase (PPA1) (1398) as upregulated in trauma is in accordance with the findings of Borregaard and Herlin, that activated neutrophils enhance the glucose consumption and consequently the production of ATP and NADPH which will be used in ROS production [23]. After trauma, the augmented level of glucagon hormone maintains blood glucose levels until depauperation. To generate energy the cells potentiate some biochemical processes as oxidation of fatty acids, which requires the pyrophosphatase enzyme (PPA1). Also, PPA1 is associated with apoptosis induction [24], consistent with the functions of other proteins described below.

Composing the trauma downregulated proteins class, ubiquitin carboxyl-terminal hydrolase L5 (UCH-L5) (1179) is a deubiquitinating enzyme and degradation of proteins by the ubiquitin-proteasome pathway is critical in regulating the levels of most cellular proteins and in the rapid elimination of misfolded proteins [25]. UCH-L5 was also described as associated with a chromatin-remodeling complex and can participate in the regulation of transcription or DNA repair [26]. The finding of this protein as downregulated after activation suggests a decrease in repair mechanisms, protein processing, ubiquitin turnover and in the regulation of transcription, as proposed by Hossain *et al.* [27].

L-plastin (354) is an actin-bundling protein which collaborates to the modifications on leukocyte cytoskeleton at membrane ruffles during activation [28]. Vimentin (521) is an intermediate filament cytoskeletal protein which participates in neutrophil adhesion and transmigration, and can be secreted to the extracellular space collaborating in cytotoxic processes, such as bacteria killing and oxidative metabolite production [29]. Beta-actin (729) is the major protein component of the cytoskeleton, can assemble into filaments and generate a motile machine capable of efficient and rapid chemotaxis in polymorphonuclear leukocytes [30]. The downregulation of these three cytoskeleton related proteins is probably related to their post-translational processing, either by secretion, proteolysis or by uptake in complexes, during the remodeling process, instead of a decreased gene expression. This confirms the findings of Suchard and Boxer [31] and Mambole *et al.* [32] who mention the cleavage of cytoskeletal proteins upon activation. The findings of Fessler *et al*. [8, 33], showing a low correlation between the transcriptome and the proteome of neutrophils are also supportive for this downregulation.

Laminin receptor 1 or RSSA (811) can function as a leukocyte surface receptor for laminin - a glycoprotein of the endothelium [34]. After exposure to cytokines in inflammatory events, the expression of laminin in endothelial cells is upregulated [35]. A study using rabbit circulating polymorphonuclear leukocytes shows laminin on their surfaces and it is presumably bound to the receptor [36], suggesting a strong binding. Our finding of fewer amounts of laminin receptor 1 after cell activation can represent an increased link between the receptor and neutrophil laminin, before the development of chemotaxis, adhesion and transmigration.

Grancalcin (1282) is a cytosolic Ca^2+^-binding protein expressed at high levels in neutrophils which translocates to the primary and secondary granules membranes upon leukocyte activation [37]. In activated neutrophils, grancalcin is related with L-plastin through Ca^2+^ dependence in adhesion and spreading processes [38] and the downregulation of both indicates a cell that is more likely to adhere and migrate, since the molecules are probably complexed (by interaction with L-plastin) in the activated state [39]. Also, S100-A9 (1543) is one of the major calcium-binding molecules expressed in neutrophils [40]. It is targeted to the cell surface upon calcium influx and is released from the leukocyte during the activation processes [41] inducing chemotaxis to other neutrophils and degranulation [42], and this is reasonable with our finding of S100-A9 downregulation. Annexin A3 (ANXA3) (1052) is a calcium-dependent phospholipid-binding protein which participates in intracellular membrane fusion and granule aggregation in neutrophils, as observed when these cells engulfed yeast particles and ANXA3 accumulated at periphagosomal area [43]. The α-soluble NSF attachment protein (SNAP) (1024) has a widespread involvement in vesicular transport pathways leading to membrane fusion among endoplasmic reticulum, Golgi apparatus and secretory vesicles, conducting to exocytosis [44]. Our findings of SNAP and ANXA3 downregulation, associated to the fact that this class of proteins is secreted from diverse cell types in exosomes [45], suggest that the activated neutrophils of those trauma patients have released these proteins.

Heat shock 70 kDa protein 1 or HSPA1A (358) can stabilize existing proteins against aggregation, protecting against oxidative damage and preventing apoptosis [46]. This protein is also considered an autoantigen chemotactic for leukocytes, released by exocytosis during leukocyte activation [47]. Such facts are coherent with the fewer amounts of this protein. Glutathione S-transferase P or GSTP1 (1336) is a cytoplasmic enzyme that plays an important role in detoxification of ROS. Reports of Yin *et al.* showed that GSTP1 protects cells against apoptosis promoted by H_2_O_2_ exposure [48].

Rho and Rac are GTP-binding proteins of the Rho family, that participates in the activity of NADPH oxidase, and regulates motile responses [49]. Rho GDP dissociation inhibitor (RhoGDI) (1262) maintains Rho protein in soluble (cytosolic) form [50]. Upon polymorphonuclear leukocyte activation, RhoGDI becomes a component of neutrophil phagosomes, suggesting a localized delivery and release of the GTPases required for Fcγ receptor-mediated phagocytosis [51]. These processes are consistent with the diminished quantities of RhoGDI found in activated neutrophils.

In the set of proteins detected only in quiescent cells, we identified a subset that participates in translation processes: KIAA0564 (71), HisRS (526), IFIT2 (595), MRI (1501), PMS2L3 (1360) and PUS7L (1479). The activity of such proteins involve nucleotide binding, gene expression modulation synthesis and processing of tRNA, DNA repair, regulation of proteasomal processing, of alternative splicing and of apoptosis. The unique presence of these six proteins in quiescent leukocytes added to the downregulation of UCH-L5 described above might be alluded to the fact that many neutrophil activation responses use constitutive biochemical pathways and enzymes performing post-translational modifications in a higher rate than new protein synthesis [52] and reinforces the hypothesis of suppressed reparative pathways in a cell inclined to programmed death. In addition, Fessler *et al.* described poor concordances between mRNA transcriptions and protein quantitation in polymorphonuclear leukocytes [8, 33].

NCK-associated protein 1-like (NCKAP1L) (102) collaborates in Rac activation, actin polymerization and myosin regulation in neutrophil chemotaxis [53]. Soon after receiving the activating stimulus, neutrophils initiate the process of shape change and movement, when complex interactions among cytoskeleton proteins, such as beta-actin and NCKAP1L, are occurring [54] and may explain its absence in the activated cell state. Heat shock 70 kDa protein 1-like (HSPA1L) (508), an allelic variant of the HSPA1A, already described as downregulated above, confirms the reduced level of this protein family, reinforcing the aspects related to oxidative damage discussed above.

Yet, desmin (603) is a filament found in neutrophils, like vimentin. It is enriched on activated neutrophils phagosomes [55] and connects to F-actin, promoting formation of pseudopods [56] and preventing its detection as unmodified.

Presenting folding similarity with albumin and vitamin D-binding protein, the albumin-like protein (679), found for the first time in neutrophils, binds to actin within the cytoskeleton [57] and to C5a, enhancing chemotaxis. After binding, the protein goes through proteolytical processing [58], what could explain its undetectable amounts in neutrophils after trauma. Zinc finger FYVE domain 19 (Zfyve19) (680), also seen for the first time in neutrophils, is a zinc binding finger protein probably involved in vesicle trafficking and phosphoinositide metabolism. The absence of Zfyve19 in activated cells could be due to its proteolytic processing after interaction with phosphatidylinositol 3-phosphate followed by recruitment to endo- and exocytical membranes [59] This finding is reinforced by the FGD1 protein, that presents a FYVE domain[60], also detected only in quiescent cells . Again, this work presents for the first time the presence of a third protein, monoamine oxidase type B (MAOB) (695), in the neutrophil. This protein localizes at the outer membrane of mitochondria and the byproducts of the reaction catalyzed by MAOB are reactive nitrogen species (RNS) and ROS, known inducers of mitochondrial damage and apoptosis [61]. MAOB disappearance after neutrophil activation could be associated to the increased free radicals generated in the granules during this leukocyte state.

In antibodies, the Fab region opsonizes the antigen, and the Fcγ region is a ligand for the Fcγ receptor, presented as FcγRIII on the quiescent polymorphonuclear leukocyte surface [7]. Upon activation of neutrophils, or during apoptosis, FcγRIII is released from the cell surface by proteolytic cleavage, and the enzyme responsible for this process is probably a membrane-bound metalloprotease released from granules [62]. The finding of antibody-dependent cellular cytotoxicity (ADCC)-enhanced Fcγ fragment (629) only on the quiescent set is coherent with neutrophil activation by trauma and consequent release of FcγRIII bound to its ligand.

In a subset of ion channel proteins, we have found CACNB2 and CLIC4. Calcium channel voltage-dependent beta-2 subunit (CACNB2) (904) allows calcium influx, participating on the activation of NADPH oxidase, adhesion, degranulation and synthesis of IL-8 [63]. The filling of intracellular Ca^+2^ stores involves physical coupling at plasma membranes of the calcium channel proteins with IP_3_ (inositol triphosphate) receptors and this fact could be the explanation for the absence of CACNB2 in activated neutrophils [64]. Chloride intracellular channel 4 (CLIC4) (982) is an anion exchanger channel for a proper cytoplasmic or phagosome acidification, through a Cl^-^/H^+^ antiporter activity [65]. Variations in intracellular pH allowed by CLIC4 modulate adhesion, chemotaxis, phagocytosis, secretion of enzymes and ROS production in neutrophils. Since chloride channels operate in neutrophils after β_2_-integrins cross linking [66], we hypothesize that CLIC4 could be undetectable in stimulated cells due to its interaction with integrins. The finding for CLIC4 is supported by the observed downregulation of GSTP1, its structural homolog.

In summary, although recent intensive study has clarified many events involving the role of neutrophils in the inflammatory response, many questions remain to be answered. Our results show differences between the proteomic patterns of quiescent and the trauma-induced activated neutrophils, with some proteins described here for the first time (albumin-like, Zfyve19 and MAOB) as associated with either quiescent or trauma-activated neutrophils. As illustrated by Fig. (**2**), among the identified proteins, a considerable subset is related to cytoskeletal reorganization, or cell motility and adhesion (L-plastin, vimentin, beta-actin, RhoGDI, NCKAP1L and desmin). Some, such as grancalcin, S100-A9, CACNB2 and CLIC4 reinforce observed correlations between motility and ion flux. Another group of identified proteins contains SNAP, ANXA3 and Zfyve19, which are involved in vesicular transport and exocytosis.

We have presented evidence that activated neutrophils may have diminished protection against oxidative damage and are prone to apoptosis, through downregulation of GSTP1, UCH-L5 and HSPA1A, upregulation of PPA1, and absence of HSPA1L and MAOB. Quantitative changes in translational and repair proteins also support this hypothesis.

## CONCLUSION

In conclusion, the results of this study allowed the identification of a set of differences between the proteomic pattern of quiescent and trauma-activated neutrophils. Some of the identified proteins are involved in the neutrophil cytoskeleton reorganization, others suggest that the neutrophil is a cell with diminished protection against oxidative damage and prone to apoptosis at the initial hours after trauma. Some proteins were described for the first time in neutrophils. Our findings may contribute to the development of laboratory and clinical strategies for controlling deleterious inflammatory responses, as seen after severe trauma and sepsis.

## Figures and Tables

**Fig. (1):**
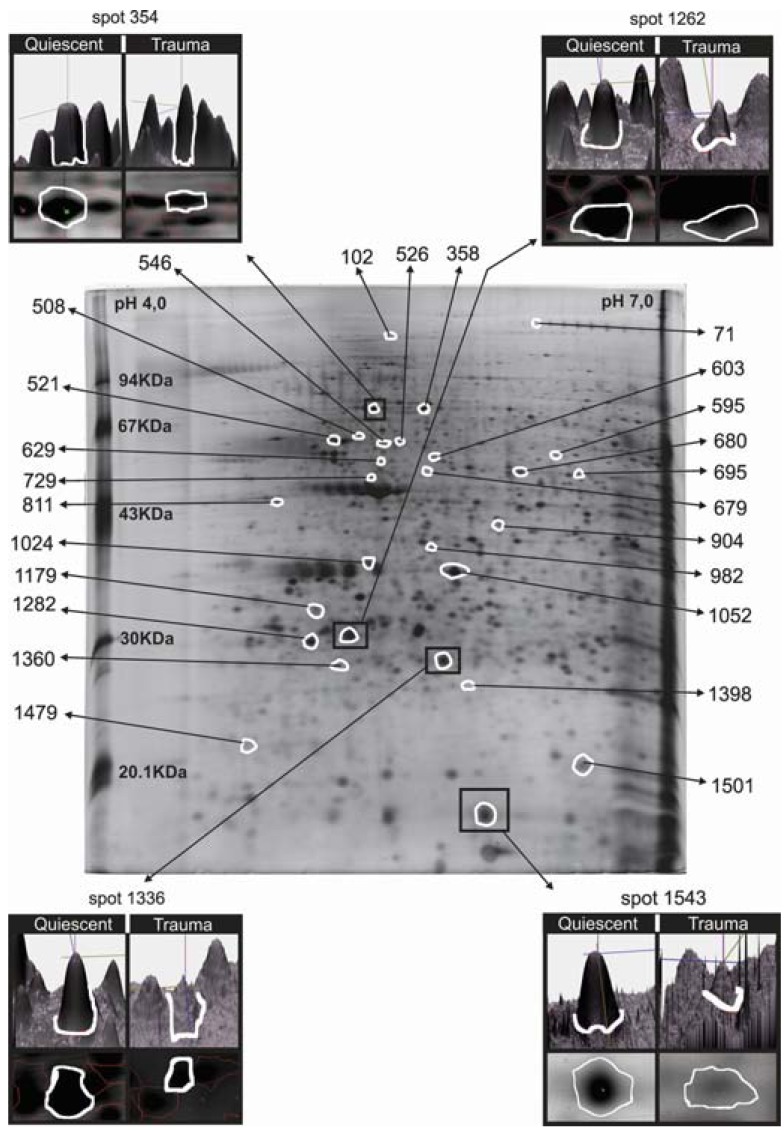
Proteomic map of neutrophils.

**Fig. (2):**
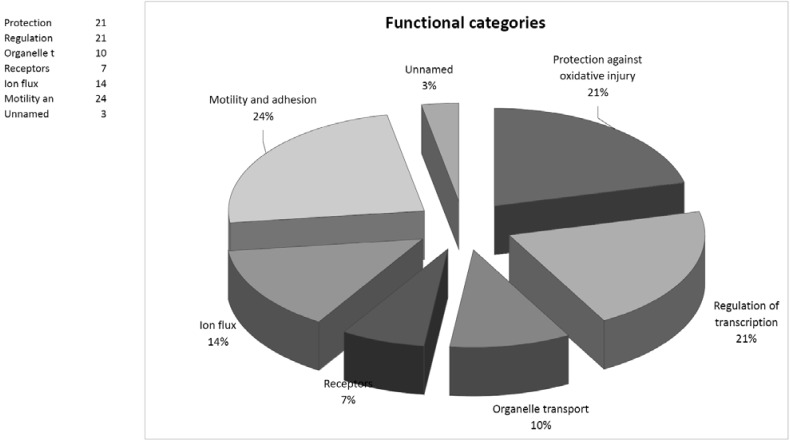
Functional categories of the 29 identified neutrophil proteins.

**Table 1.** Neutrophil proteins identified by MALDI-TOF PMF and MS/MS fragmentation. Spot numbers coincide with Fig. (1)

Spot	Expression Parameter^a^	p-value	Accession Number	Name	Activity	pI (theor/exp)	Mr (theor/exp)(kDa)	PMF Score^b^	PMF C(%)^c^	MS/MS Score^d^	MS/MS peptides
71	Q		gi|3043652	KIAA0564	Translation	6.37 / 6.56	163 / 176	354	39	-	
102	Q		gi|34485727	NCKAP1L	Structural / Motility	6.39 / 5.53	130 / 120	97	19	-	
354	↓T (4.59)	0.003	gi|1346733	L-plastin	Structural / Cytoskeleton remodelling	5.20 / 5.20	71 / 70	259	64	138	R.NWMNSLGVNPR.V K.MINLSVPDTIDER.T K.FSLVGIGGQDLNEGNR.T
358	↓T (3.92)	0.024	194248072	HSPA1A	Protein metabolism / Oxidative protection	5.48 / 5.56	70 / 75	361	60	67	R.TTPSYVAFTDTER.L R.ARFEELCSDLFR.S K.NQVALNPQNTVFDAKR.L
508	Q		gi|124256496	HSPA1L	Protein metabolism / Oxidative protection	5.76 / 5.21	71 / 66	88	20	-	
521	↓T (5.28)	0.003	gi|62414289	vimentin	Structural / Motility	5.06 / 5.03	54 / 62	201	49	71	R.SLYASSPGGVYATR.S R.TYSLGSALRPSTSR.S R.ISLPLPNFSSLNLR.E K.FADLSEAANRNNDALR.Q
526	Q		gi|62766469	HisRS	Translation	5.72 / 5.51	58 / 62	181	46	-	
546	Q		gi|194389088 P98174.2	FGD1	Granule transport	5.57 / 5.32	60 / 58	91	22	-	
595	Q		gi|153082755	IFIT2	Translation/apoptosis	6.32 / 6.59	55 / 59	82	23	-	
603	Q		gi|1408188	desmin	Structural/motility	5.21 / 5.70	53 / 60	238	60	-	
629	Q		gi|171848866	ADCC-Enh. Fc Fragment	Cell stimulation	6.37 / 5.35	26 / 54	78	60	39	R.VVSVLTVLHQDWLNGK.E
679	Q		gi|763431	albumin-like	Structural	5.69 / 5.66	53 / 56	162	46	96	K.KVPQVSTPTLVEVSR.N R.RHPDYSVVLLLR.L K.DVFLGMFLYEYAR.R
680	Q		gi|119612854	Zfyve19	Granule transport	5.21 / 6.13	48 / 53	109	27	-	
695	Q		gi|38202207	MAOB	Amine metabolism/oxidative protection	7.20 / 6.79	59 / 52	103	34	-	
729	↓T (2.74)	0.027	gi|14250401	b-actin	Structural/motility	5.56 / 5.27	41 / 49	80	31	63	R.AVFPSIVGRPR.H K.SYELPDGQVITIGNER.F
811	↓T (2.67)	0.040	gi|125969	RSSA	Adhesion receptor	4.79 / 4.69	33 / 46	192	53	212	K.FAAATGATPIAGR.F R.AIVAIENPADVSVISSR.N R.FTPGTFTNQIQAAFREPR.L
904	Q		gi|150039531	CACNB2	Voltage-gated calcium channel	6.41 / 6.16	46 / 43	84	31	-	
982	Q		gi|7330335	CLIC4	Voltage-gated chloride channel	5.45 / 5.60	29 / 38	76	41	-	
1024	↓T (5.76)	0.003	gi|116242794	SNAP	Granule transport	5.23 / 5.23	34 / 33	197	75	-	
1052	↓T (6.34)	0.001	gi|4826643	ANXA3	Structural/Granule transport	5.63 / 5.63	37 / 36	268	62	499	K.ALLTLADGR.R K.LTFDEYR.N M.ASIWVGHR.G + Acetyl (Protein N-term) R.NTPAFLAER.L K.GIGTDEFTLNR.I K.GAGTNEDALIEILTTR.T K.GELSGHFEDLLLAIVNCVR.N
1179	↓T (2.47)	0.006	gi|55859537	UCH-L5	Protein metabolism/DNA repair	4.95 / 4.88	29 / 32	78	52	-	
1262	↓T (10.66)	0.011	gi|56676393	Rho GDI	Motility/NADPH oxidase assembly	5.10 / 5.12	23/ 29	167	81	38	K.YVQHTYR.T
1282	↓T (8.29)	0.001	gi|17943195	Grancalcin	Structural/Motility	5.77 / 4.87	19 / 28	69	28	33	R.ALTDFFR.K R.CLTQSGINGTYSPFSLETCR.I
1336	↓T (2.53)	0.003	gi|119578233	GSTP1	Oxidative protection	5.43 / 5.67	23 / 28	143	52	247	K.FQDGDLTLYQSNTILR.H K.ALPGQLKPFETLLSQNQGGK.T
1360	Q		gi|119592171	PMS2L3	Translation/DNA repair	4.52 / 5.13	19 / 27	67	25	-	
1398	↑T (0.38)	0.050	gi|119626587	PPA1	Energy metabolism	6.82 / 6.03	16 / 25	70	50	-	
1479	Q		gi|119603770	PUS7L	Translation	4.57 / 4.49	28 / 22	70	26	-	
1501	Q		gi|208022703	MRI	Post-translational processing	5.16 / 6.50	17 / 20	90	49	-	
1543	↓T (4.97)	0.008	gi|115444	S100-A9	Motility	5.71 / 5.71	13 / 13	83	52	-	

a expression parameter: comparison between quiescent and trauma activated cells  Q: Detected only in quiescent neutrophils  ↓ T: Downregulated in trauma conditions  ↑ T: Upregulated in trauma conditions  The number in parenthesis indicates the ratio for the non-exclusive spots

b PMF Score: search score form PMF analysis using Mascot software and the NCBI non-redundant database.

c PMF C (%): percentage of sequence coverage by matched peptides used in PMF searches.

d MS/MS score: search score from peptide fragmentation spectra analysis using Mascot software and the NCBI non-redundant database.
